# TLR5 is a new reporter for triple‐negative breast cancer indicated by radioimmunoimaging and fluorescent staining

**DOI:** 10.1111/jcmm.14707

**Published:** 2019-10-01

**Authors:** Dai Shi, Weiwei Liu, Shanshan Zhao, Chao Zhang, Ting Liang, Guihua Hou

**Affiliations:** ^1^ Key Laboratory for Experimental Teratology of the Ministry of Education and Biomedical Isotope Research Center School of Medicine Shandong University Jinan China

**Keywords:** fluorescence imaging, Phosphorautoradiography, Radioiodine 125, TLR5, triple‐negative breast cancer

## Abstract

Triple‐negative breast cancer (TNBC) is a highly aggressive tumour that lacks marker for targeted diagnosis. Recently, it was reported that toll‐like receptor 5 (TLR5) was associated with some kind of tumours, especially in TNBC, but whether it could be used as a non‐invasive monitoring target is not fully understood. Here, we established TLR5^−^ 4T1 cell line with lentivirus‐shRNA‐TLR5 knock‐down transfection (with tag GFP, green fluorescent protein, TLR5^−^ 4T1) and control TLR5^+^ 4T1 cell line with negative control lentivirus transfection. The effect of TLR5 down‐regulation was detected with qPCR and Western blot. ^125^I‐anti‐TLR5 mAb and control isotype ^125^I‐IgG were prepared and injected to TLR5^+/−^ 4T1‐bearing mice models, respectively. Whole‐body phosphor‐autoradiography, fluorescence imaging and biodistribution were performed. Furthermore, ex vivo tumour TLR5 expression was proved through immunohistochemistry staining. We found that ^125^I‐anti‐TLR5 mAb could bind to TLR5^+^ 4T1 with high affinity and specificity. Whole‐body phosphor‐autoradiography after ^125^I‐anti‐TLR5 mAb injection showed TLR5^+^ 4T1 tumour images in 24 hours, more clearly in 48 hours. Radioactivities in tumour tissues were positively related with TLR5 expression. Biodistribution assay showed that ^125^I‐anti‐TLR5 mAb was mainly metabolized through the liver and kidney, and ^125^I‐anti‐TLR5 mAb was much more accumulated in TLR5^+^ 4T1 tumour than TLR5^−^ 4T1. In vivo fluorescence imaging successfully showed tumour tissues clearly both in TLR5^+^ and TLR5^−^ 4T1 mice compared with lentivirus untreated 4T1 tumour. Immunohistochemistry staining showed that TLR5 expression in tumours was indeed down‐regulated in TLR5^−^ 4T1 mice. Our results indicated that ^125^I‐antiTLR5 mAb was an ideal agent for non‐invasive imaging of TLR5^+^ tumours; TLR5 may be as a novel molecular target for TNBC non‐invasive diagnosis.

## 1 INTRODUCTION

Breast cancer is the most common cancer with high mortality in female worldwide, which can be classified into two groups, triple‐negative breast cancer (TNBC) and non‐TNBC.^1^, ^2^, ^3^ TNBC accounts for 12%‐24%, more aggressive and more prone to metastasis. Currently, clinical targeting management of breast cancer mainly depends on molecular markers: oestrogen receptor (ER), progesterone receptor (PR) and human epidermal growth factor receptor 2 gene (HER2). These molecules could provide therapeutic predictive and prognostic indicators. However, since that TNBC does not express ER, PR and HER2, targeted diagnosis and therapy are not available for this kind of breast cancer.^4^, ^5^ Early diagnosis is critical and urgently needed for improving prognosis of TNBC patients.

Nuclear medicine molecular imaging is a kind of non‐invasive whole‐body scanning modality based on metabolism and function abnormal, more suitable for the detection and quantification of tumour target molecular, compared with traditional imaging CT (computed tomography) and MRI (magnetic resonance imaging) in diagnosis and tumour staging.^6^ Nowadays, ^18^F‐FDG (F‐18‐deoxyglucose) PET/CT (positron emission tomography/computed tomography) was widely used in clinic for evaluation tumour metabolisms.^7^ However, ^18^F‐FDG is a non‐specific agent, which made diagnosis sometimes not accurate. Searching the better molecular target for tumour diagnosis and therapy is critical for clinic.

Recently, many reports showed that innate molecules were participated in tumour developments and prognosis, such as CCAT2 (a kind of long non‐coding RNA, metastasis and prognosis)^8^ and three miRNAs (potential prognostic biomarker in patients with bladder cancer).^9^ Molecular imaging developed much quickly, which showed great potential in tumours early diagnosis and treatment based on the change of physical and metabolisms.^10^ Among them, toll‐like receptors (TLRs) caught great attention. TLRs are membrane‐bound proteins, expressed originally mainly in immunocytes**,** such as monocyte/macrophage, vascular endothelial cells,^11^ and also expressed on other kinds of cells, such as cancer cells. TLR family (TLR1‐13) recognizes different pathogen‐associated molecular patterns (PAMPs).^12^, ^13^, ^14^, ^15^ As a member of TLRs, TLR5 is known to specifically recognize flagellin, which is the only protein ligand in TLR family.^16^ Recently, TLR5 was found highly expressed on a variety of cancer, such as ovarian cancer,^17^ gastric cancer 18 and colon cancer,^19^ and tumours' growth was significantly inhibited by bacterial flagellin activation through TLR5 pathway.^21^ More importantly, TLR5 was found highly expressed in both TNBC and non‐TNBC,^20^ tightly related to cancer progression, while TLR5 expressed on immunocytes or tumour cells within breast cancer existed controversies. Considering of TLR5 expression co‐related with cancer progression, we postulated that TLR5 may be as a predictor of TNBC development**,** so we choose TNBC cell line to investigate whether it could be a target molecule of non‐invasively diagnosis for TNBC.

For breast cancer molecular imaging, there were some reports about target molecules with labelled radioisotope, for example ^18^F‐labelled aptamers of human epidermal growth factor receptor 2 (Her2/ErbB2),^21^ ^125^I/^131^I‐labelled anti ICAM‐1^22^ ( intercellular cell adhesion molecule‐1) and ^89^Zr‐Transferrin.^23^ However, these target molecules still exist some deficiency, such as low selection and low molecular expression on tumour. Our laboratory has successfully prepared iodine 131 labelled anti‐TLR5 antibody and it could be used to indicate allotransplantation rejection.^24^ However, whether it could be used as a non‐invasive monitoring target for TNBC is not fully understood. Here, we constructed a TLR5 gene knock‐down TNBC cell line 4T1 with lentivirus‐shRNA knock‐down virus transfection and prepared TLR5 targeting radioisotope labelled probe, to investigate whether TLR5 could be a new target for TNBC non‐invasive monitoring in vivo and underlying mechanisms.

## 2 MATERIALS AND METHODS

### 2.1 Cell culture and Lentivirus‐shRNA‐TLR5

The murine triple‐negative breast cancer (TNBC) cell line 4T1^25^ was obtained from the American Type Culture Collection (ATCC). Cell was cultured in RPMI 1640 medium supplemented with 10% foetal bovine serum (FBS; Invitrogen) and 1% penicillin/streptomycin (Hyclone, USA) at 37°C in a humidified incubator with 5% CO_2_. The lentivirus‐shRNA for TLR5 knock‐down and negative control virus was purchased from GenePharma.

### 2.2 In vitro transfection

4T1 cells were plated in 24‐well plates (1 × 10^5^/well) overnight, then discarded the supernatant, replaced with virus dilution(negative control virus and lentivirus‐shRNA TLR5 were mixed with the fresh medium in a ratio of 1 to 100, respectively) and then incubated at 37°C in a humidified incubator with 5% CO_2_ for 24 hours. Finally, replaced the virus dilution with fresh medium and continued to incubate at 37°C in a humidified incubator with 5% CO_2_ for another 48 hours. Then, we calculated the transfected efficiency. Puromycin was used to filter out successfully transfected cells. If the transfection is successful, the cells will express green fluorescent protein (both of negative control virus and lentivirus‐shRNA TLR5).

### 2.3 Identification of TLR5 expression

TLR5 expression was determined by qPCR and Western blot.

For qPCR, total RNA was extracted from 4T1 cells using the TRIzol reagent (Invitrogen) according to the instructions of manufacturer and then determined RNA concentration. cDNA first strand was synthesized from RNA using TransScript First‐Strand cDNA Synthesis SuperMix (TRANSGEN BIOTECH). Finally, mRNA was measured using TransStart Tip Green Qpcr SuperMix (TRANSGEN BIOTECH). TLR5 quantitative primers: 5′‐GCAGGATCATGGCATGTCAAC‐3′(forward) and 5′‐ATCTGGGTGAGGTTACAGCCT‐3′(reverse).

For Western blot (WB), cells were harvested at about 70% confluency. Total protein concentration was measured using a protein assay kit (Beyotime Biotechnology). Twenty micro gram of total protein was loaded into Bio‐Rad gel (Bio‐Rad Laboratories), along with Chameleon Duo ladder protein marker (SMOBIO). Gel electrophoresis was performed at 80 mV for 30 minutes and then up to 100 V for 60 minutes. All proteins were then transferred to a nitrocellulose membrane at 200 mA for 100 minutes. The membrane was blocked with skim milk (5%) blocking buffer for 2 hours at room temperature and further incubated with a rabbit antimouse TLR5 mAb (1:1000, dilution, Abcam, UK) and rabbit antimouse GAPDH mAb (1:10 000 dilution, Abcam, UK) overnight at 4°C. Next, the membrane was washed three times with TBS with Tween 20 (TBS‐T) and incubated with anti‐rabbit secondary antibodies (1:10 000 dilution, Abcam, UK) for 1 hours at room temperature. The washed membrane was scanned and quantitatively analysed by Tanon 4200 imaging system.

### 2.4 Evaluation of viability for TLR5 knock‐down 4T1 cells

CCK‐8 assay was used to evaluate its proliferation. Furthermore, flow cytometry was used to detect apoptosis. For CCK‐8, TLR5^+/−^ 4T1 cells (3 × 10^3^/well) were respectively plated in 96‐well plates overnight, and then microplate reader was used to measure the absorbance at 450 nm at 0, 6, 24 and 48 hours.

For apoptosis, the TLR5^+/−^ 4T1 cells were respectively digested with 0.25% trypsin (no EDTA and no phenol red) and then centrifuged to collect the cells. The cells were washed twice with cold PBS, suspended in 400 μL of 1× Annexin V binding solution at a concentration of approximately 1 × 10^6^ cells/mL, added 5 μL of Annexin V‐YF647A and 10 μL of PI staining solution, mixed gently and incubated at 4°C for 15 minutes in the dark and filtered with a 200‐mesh filter and immediately detected by flow cytometry.

### 2.5 Preparation of ^125^I‐anti‐TLR5 mAb/ ^125^I‐IgG

Briefly, 10 μg of anti‐TLR5 mAb/IgG was added to 100 μL of 0.05 M phosphate buffered (PB, pH 7.4), followed by the addition of 11.1 MBq (300 μCi) of Na^125^I, the mixture was incubated for 20 minutes at room temperature, and then the reaction was stopped by adding 150 μL of 0.05 M pH 7.4 PB and incubated for another 10 minutes at room temperature.^24^ The labelled compound was purified with PD‐10 Sephadex G‐25 (GE Healthcare), and the labelled rate was calculated. The mixture of 0.9% saline and methanol with a volume ratio of 1:2 was used as an unfolding agent. The stability of ^125^I‐anti‐TLR5 mAb and ^125^I‐IgG was determined in PBS and serum.

### 2.6 Binding assay of ^125^I‐anti‐TLR5 mAb

TLR5^+/−^ 4T1 cells were seeded in a 24‐well plate at 5 × 10^5^ cells per well, respectively. ^125^I‐antiTLR5 mAb in PBS solution (0.01M, pH 7.4, 1‐30 nmol/L) was added into the plates, then incubated at room temperature for 2 hours and discarded the supernatant, and cells were washed twice with iced 1 × PBS (0.01M, pH 7.4, containing 0.1% BSA), then harvested and determined radioactivities with a gamma counter. Non‐specific binding was evaluated by the presence of non‐labelled anti‐TLR5 mAb (diluted in 0.01 M, pH 7.4 PBS, 1–30 μmol/L) in the same wells. The binding results including maximum binding ability (B_max_) and dissociation constant (K_d_) were obtained through GraphPad Prism software. For competitive binding, TLR5^+^ 4T1 cells were seeded in a 24‐well plate at 5 × 10^5^ cells per well. 0.1‐1000 nmol/L anti‐TLR5 mAb and 10 nmol/L ^125^I‐anti‐TLR5 mAb were used. The mixture was incubated at 37°C for 45 minutes and finally discarded the supernatant, and cells were washed twice with iced 1 × PBS containing 0.1% BSA, then harvested and the radioactivity determined with a gamma counter.

### 2.7 Animal model

All animal studies were conducted in accordance with protocols approved by the Animal Care and Use Committee of Shandong University, China. For tumour‐bearing mice models, subcutaneous tumours of TLR5^+^ and TLR5^−^ 4T1 cell lines were induced in 5‐week‐old male nude mice. TLR5^+^ 4T1 Tumour cells (2 × 10^6^) suspended in 200 μL PBS (pH7.4, 0.01 M) were subcutaneously injected into their lower right flank. TLR5^−^ 4T1 Tumour cells (2 × 10^6^) suspended in 200 μL PBS were subcutaneously injected into their lower left flank. Mice were monitored every other day. The animals were used for phosphor‐autoradiography imaging, fluorescence imaging and biodistribution once the tumour had reached about 10 mm in diameter.

### 2.8 Dynamic Whole‐body phosphor‐autoradiography

Three days before injection of radiotracers, 4% potassium iodide was added to the drinking water to block the thyroid gland uptake of iodine. Tumour‐bearing mice (n = 5 per group) were injected for each with 0.37 MBq of ^125^I‐antiTLR5 mAb (0.38μg) or ^125^I‐IgG (0.38μg) through tail vein. For blocking group, unlabelled anti‐TLR5 mAb (100 μg) was injected 30 minutes before ^125^I‐antiTLR5 mAb injection. Phosphor‐autoradiography scanning was conducted at 24, 48 and 72 hours after injection. 0.6% sodium pentobarbital was used for anaesthesia. Anaesthetized mice were placed on the storage phosphor screen plate (back to the plate). After 20 minutes in the dark, the plate was immediately covered with an opaque plastic sheet and then transferred to the Cyclone Plus scanner (PerkinElmer Life Sciences). Semi‐quantitative analysis was performed by manually drawing rectangular regions of interest (n = 5) within the target area at each time‐point. Digital light units (DLU)/mm^2^ were obtained using OptiQuant™ image analysis software 5.0 (PerkinElmer Life Sciences). In addition, we stripped the tumour separately and performed the imaging.

### 2.9 Fluorescence imaging

4T1 cells transfected with lentivirus‐TLR5 knock‐down and negative control lentivirus express green fluorescent protein (GFP) after integrated into 4T1 cells. Subcutaneous tumours of the TLR5^−^ and TLR5^+^ 4T1 cells were induced described as above. TLR5^+^ 4T1 on lower right flank and TLR5^−^ 4T1 on lower left flank and the 4T1 cells without lentivirus transfected on back. When tumour reached 10 mm in diameter, anaesthesia was performed, then surface skin of the tumour was peeled, and the mouse was placed on the imaging plate and photographed (Belly down).

### 2.10 Biodistribution studies

Three days before injection of radiotracers, 4% potassium iodide was added to the drinking water to block the thyroid gland uptake of iodine. ^125^I‐anti‐TLR5 mAb or ^125^I‐IgG (0.37 MBq) was injected into model mouse (n = 5 per group), respectively, and mice were killed and dissected at 24, 48 and 72 hours. The tumours, blood and major tissues/organs (heart, lung, liver, kidney, spleen, small intestine and muscle) were harvested and weighed. Samples and primed standards were measured with gamma counter. Tissue radioactivity is expressed as the per cent injected dose per gram (%ID/g). The target‐to‐non‐target ratio was defined as the tumour‐to‐opposite‐muscle radioactivity (T/NT) ratio.

### 2.11 H&E and immunohistochemistry staining

Tumour‐bearing mice (n = 5) were executed at 72 hours after whole‐body phosphor‐autoradiography finished. Tumours were harvested for immunohistochemical staining with rabbit polyclonal TLR5 antibody (Biosynthesis Biotechnology Co., Ltd.) and DAB chromogen (Biogenics, Napa). Immunohistochemistry was performed with SP‐9002 Histostain™ Plus kits (ZSGB‐BIO) according to the manufacturer's protocols. The slides were visualized at a magnification of 200× and 400×. Corresponding positive areas of slides were analysed （five fields per slides） by the Image‐Pro Plus software version 4.5.0.29 (Media Cybernetics).

### 2.12 Statistical analysis

The data are presented as the means ± standard deviation from three independent experiments. Student's *t* test was used by GraphPad Prism version 5 software (GraphPad Software, Inc). Significant difference was indicated by ^*^ *P* < .05, ^***^ *P* < .01. SAS version 9 (SAS Institute Inc) and was used for statistical analyses.

## 3 RESULTS

### 3.1 Lentivirus transfection and TLR5 expression

**Figure 1:**
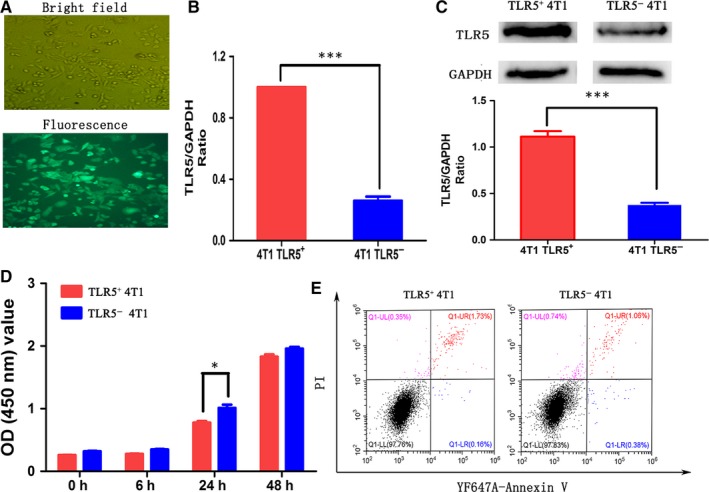
TLR5‐knock‐down lentivirus transfection in 4T1 cell lines and effect on 4T1 cell viability. TLR5‐knock‐down lentivirus transfection efficiency in 4T1 cells (A). The TLR5 mRNA expression between TLR5 ^+^ and TLR5^‐^ 4T1 cells was detected by qPCR (n = 3, ^***^ *P* < .01) (B). The TLR5 protein expression between TLR5^+^ and TLR5^−^ 4T1 cells was detected by Western blot (n = 3, ^***^ *P* < .01) (C). CCK8 assay showed TLR5^−^ 4T1 cells had higher proliferation ability than TLR5^+^ 4T1 cells (n = 3, ^*^ *P* < .05) (D). Flow cytometry showed there was no difference for cell apoptosis between TLR5^+^ and TLR5^−^ 4T1 cells (n = 3, *P* > .05) (E).The data are presented as the means ± SD from three independent experiments. The data were analysed by Student's *t* test. ^*^ *P* < .05, ^***^ *P* < .01

Lentivirus transfection efficiency is shown in Figure 1A, and transfection efficiency reached to 100%. 4T1 cells expressed TLR5 as shown in Figure 1B (qPCR) and Figure 1C (WB). The expression of TLR5 was apparently knocked down in TLR5^−^ 4T1 cells compared with negative virus transfected 4T1 cells (*P* < .05) and also lower than no virus transfected 4T1 cells (*P* < .05. data not shown).

### 3.2 Effect of TLR5 down‐regulation on viability

CCK‐8 assay showed that the proliferation ability of TLR5^−^ 4T1 cells was apparently higher than TLR5^+^ 4T1 cells which indicated that TLR5 may down‐regulate the 4T1 cell proliferation (Figure 1D). In addition, apoptosis in TLR5 down‐regulated 4T1 cells was not obviously changed compared with negative control group (Figure 1E, *P* > .05).

### 3.3 Successful preparation of ^125^I‐anti‐TLR5 mAb and ^125^I‐IgG

**Figure 2:**
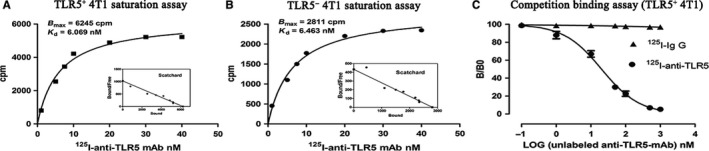
In vitro evaluation of prepared radiolabelled tracers. Representative saturation binding curve and Scatchard plots of ^125^I‐anti‐TLR5 mAb binding to TLR5^+^ 4T1 cells (A) and TLR5‐ 4T1 cells (B). The concentration of the labelled tracer (^125^I‐anti‐TLR5 mAb or ^125^I‐IgG) was kept constantly, and increasing concentrations of unlabelled anti‐TLR5 mAb were used to compete with the ^125^I‐anti‐TLR5 mAb binding (C)

As shown in Figure 2A and 2, we identified a single‐affinity sites in TLR5^+^ 4T1 and TLR5^−^ 4T1 cells. For TLR5^+^4T1 vs TLR5^−^ 4T1, a Scatchard plot and computer curve fitting of the saturation data revealed a K_d_ value of 6.069 vs 6.463 nmol/L and a B_max_ value of 124 vs 56.22 cpm/10^4^ cells. The results suggested that even total TLR5 expression decreased in TLR5^−^ 4T1 cells, and the affinity between ^125^I‐anti‐TLR5 mAb and TLR5 was not changed apparently in two groups. Labelling rate was 95.90 ± 0.60% for ^125^I‐anti‐TLR5 mAb and 92.62 ± 0.34% for ^125^I‐IgG. The radiochemical purity of ^125^I‐antiTLR5 mAb and ^125^I‐IgG were both greater than 95%, respectively. Radiochemical purity of ^125^I‐anti‐TLR5 and ^125^I‐IgG were more than 90% up to 72 hours, relatively stable in serum and NS, and no significant difference between them was detected. Competitive binding analysis with excess unlabelled anti‐TLR5 mAb (>500‐fold) could almost completely block the binding of ^125^I‐anti‐TLR5 mAb (<5%), while only about 3% non‐specific binding observed for ^125^ I‐IgG (Figure 2C).

### 3.4 Dynamic Whole‐body phosphor‐autoradiography and Fluorescence imaging

**Figure 3:**
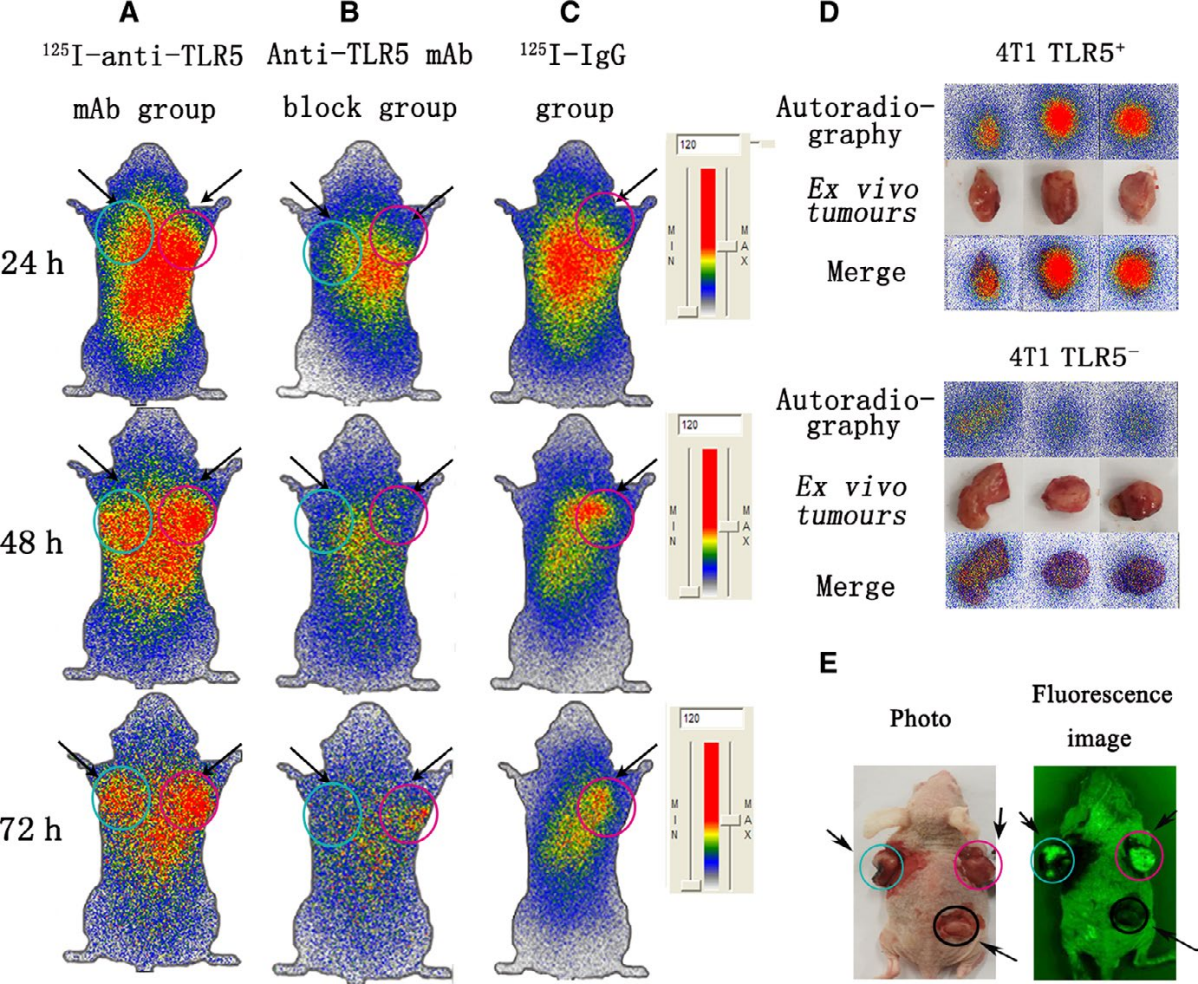
Whole‐body phosphor‐autoradiography and fluorescence imaging. Representative images were obtained at 24, 48 and 72 h post‐injection of ^125^I‐anti‐TLR5 mAb and showed apparently radioactivities in TLR5^+^ 4T1 tumour (A). Representative images showed no significant radioactivity accumulation TLR5^+^ 4T1 tumour at 24, 48 and 72 h post‐injection of ^125^I‐antiTLR5 mAb in block group (30 min prior to injection of ^125^I‐anti‐TLR5 mAb, 100 μg anti‐TLR5 mAb in 100 μL was injected through the tail vein （B). Representative images were obtained at 24, 48 and 72 h post‐injection of ^125^I‐IgG (C). Representative images of isolated tumours (isolated from model mice in Figure 3A at 72 h) (D). Representative fluorescence image of 4T1 tumour‐bearing mouse model (E).For A, B, C and E, the arrow on the left referred to the TLR5‐ tumour (blue circle), and the right is TLR5^+^ tumour (pink circle). For E, the bottom arrow referred to the TLR5^+^ tumour (without lentivirus transfection) (black circle). The data are from three independent experiments

Whole‐body phosphor‐autoradiography was performed at 24, 48 and 72 hours post‐injection of two tracers to tumour‐bearing mice separately. The radioactivities uptake in tumour increased from 24 hours and declined at 72 hours after tracer injection (Figure 3A). TLR5^+^ 4T1 tumours exhibited higher uptake, while TLR5^−^ 4T1 tumours showed lower tumour uptake at all checking time‐points. Radioactivity DLU (digital light units)/mm^2^ of tumour area reached 121 042 ± 5587 cpm for TLR5^+^ 4T1 tumour, whereas only 34 245 ± 2747 cpm for TLR5^−^ 4T1 tumour. Block group (Figure 3B) and ^125^I‐IgG (Figure 3C) group showed no obvious tumour image at any time‐point, which suggested the specific accumulation of ^125^I‐anti‐TLR5 mAb in TLR5‐positive tumour. For isolated tumour ex vivo imaging, TLR5^+^ 4T1 tumours showed much higher uptake radioactivities than TLR5^−^ 4T1 tumours (Figure 3D). In order to show the green florescent better, the surface skins of the tumours were stripped. As shown in Figure 3E, the 4T1 tumours on left side was transfected by lentivirus‐TLR5 knock‐down, 4T1 tumour on right side was transfected with negative control lentivirus and on the back one was lentivirus‐non‐treated 4T1 tumour. Tumours (green colour) on both sides of nude mice were showed clearly, while the 4T1 tumour without lentivirus transfected appeared black and no green colour. More importantly, we found that compared with phosphor‐autoradiography imaging, we could more clearly visualize tumour location and edge with green fluorescence labelled tumour cells, while TLR5^+^ tumour without virus transfected only showed radioactivities accumulation. The results indicated that double image with radioisotope and florescence could further confirm that TLR5 was a good reporter for TNBC.

### 3.5 Biodistribution studies

**Figure 4:**
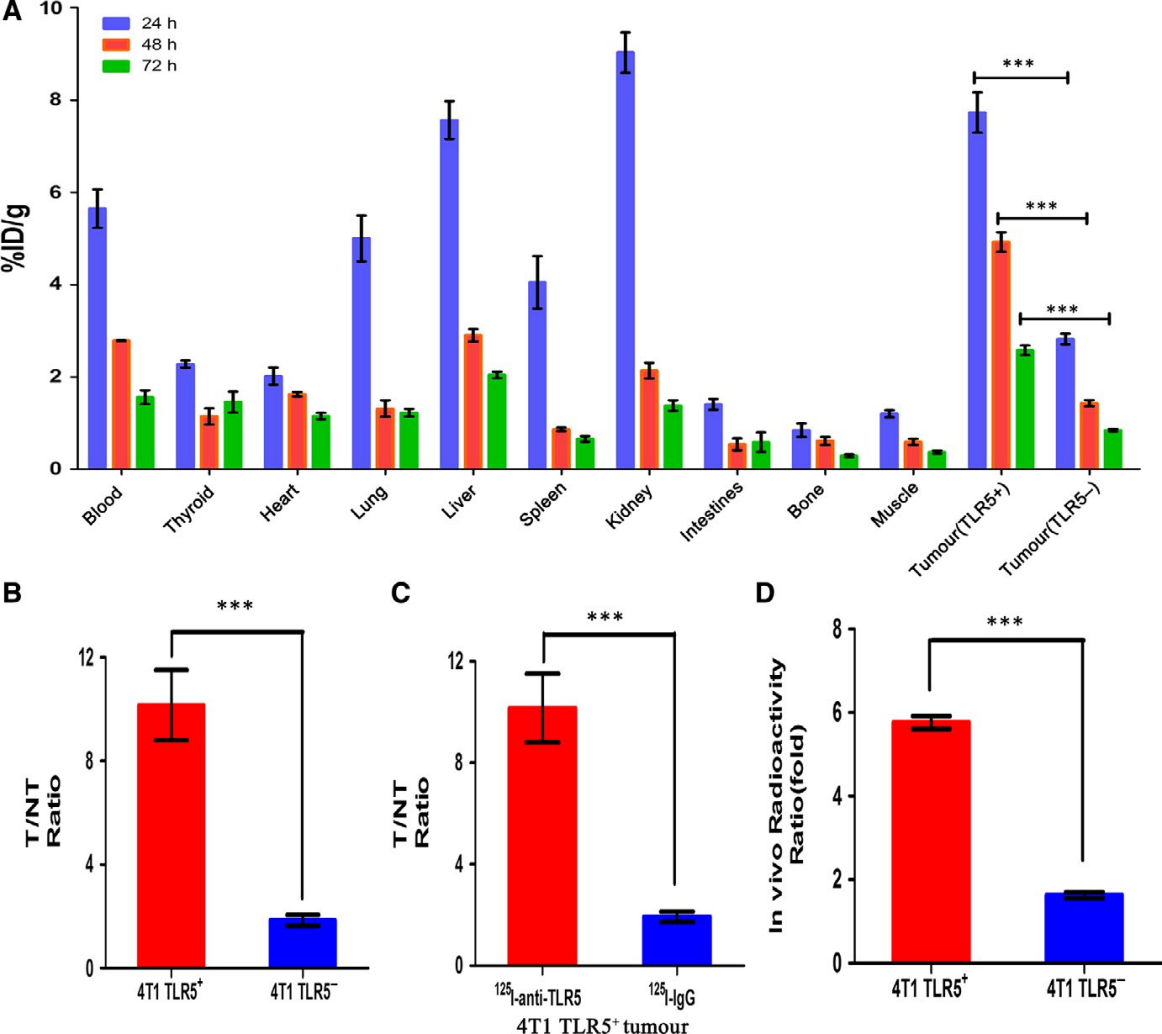
Ex vivo biodistribution studies. Biodistribution of two tracers at 24, 48 and 72 h after tracer injection in different organ or tissues. Tissue radioactivity is expressed as %ID/g, the per cent injected dose per gram (A). The radioactivity ratio of target (tumour) to NT (non‐target, opposite muscle) for TLR5^+/−^ 4T1 cells bearing mice injected with ^125^I‐anti‐TLR5 at 48 h (B). Comparison of TLR5^+^ 4T1 cells bearing mice injected with ^125^I‐anti‐TLR5 and ^125^I‐ IgG at 48 h (C). Comparison of in vivo radioactivity ratio (DLU/mm^2^, digit light units per mm^2^ from tumour and opposite same quantity area) of TLR5^+/−^ 4T1 tumours in whole‐body phosphor‐autoradiography at 48 h (D). n = 5, ^***^ *P* < .01. The data are presented from three independent experiments. The data were analysed by Student's *t* test. ^*^ *P* < .05, ^***^ *P* < .01

Ex vivo biodistribution studies were performed to validate the imaging results and to further quantify the ^125^I‐antiTLR5 mAb uptake at 24, 48 and 72 hours in the TLR5^+/−^ 4T1 tumour‐bearing model group (Figure 4A). The results revealed that TLR5^+^ tumour had the highest T/NT ratio at the 48 hours. Other tissues with higher uptake were the liver, kidney and lung. All other tissues (include TLR5^−^ tumour) had a lower %ID/g, which was in agreement with the imaging data. As shown in Figure 4A, ^125^I‐antiTLR5 mAb exhibited higher targeting efficiency in TLR5^+^ 4T1 tumours, compared with TLR5^−^ 4T1 tumours. The uptake of ^125^I‐antiTLR5 mAb in TLR5^+^ 4T1 tumours at 24, 48 and 72 hours post‐injection was 7.725 ± 0.7525, 4.9225 ± 0.36 and 2.5775 ± 0.1825 (%ID/g), T/NT ratio of 6.481 ± 0.6023, 8.413 ± 0.5270 and 7.152 ± 1.040, respectively, while the uptake of ^125^I‐antiTLR5 mAb in TLR5^−^ 4T1 tumours was 2.8225 ± 0.1975, 1.4325 ± 0.1125 and 0.845 ± 0.0475 (%ID/g), with T/NT ratio of 2.353 ± 0.091, 2.489 ± 0.1541 and 2.308 ± 0.1631. The T/NT ratio of ^125^I‐antiTLR5 mAb group in TLR5^−^ 4T1 tumours was significantly lower than in TLR5^+^ 4T1 tumours group, *P* < .05 (Figure 4B). Compared with ^125^I‐antiTLR5 mAb group,the T/NT ratio of ^125^I‐IgG group was only 2.023 ± 0.2149 at 48 hours in same TLR5^+^ 4T1 tumour, suggesting the non‐specific tumour binding of ^125^I‐IgG (Figure 4C). In block group, the uptake of ^125^I‐anti‐TLR5 mAb was only 1.57 ± 0.13%ID/g at 48 hours, suggesting the specificity of ^125^I‐antiTLR5 binding in vivo. Other organs and tissues displayed minimal or background radioactivity levels, in agreement with the imaging data. And we also quantified our in vivo phosphor‐autoradiography result in Figure 4D, which showed higher radioactivity ratio in TLR5^+^4T1 tumours than TLR5^−^ 4T1 tumours at 48 hours after tracer injection (*P* < .05).

### 3.6 H&E and immunohistochemistry staining

**Figure 5:**
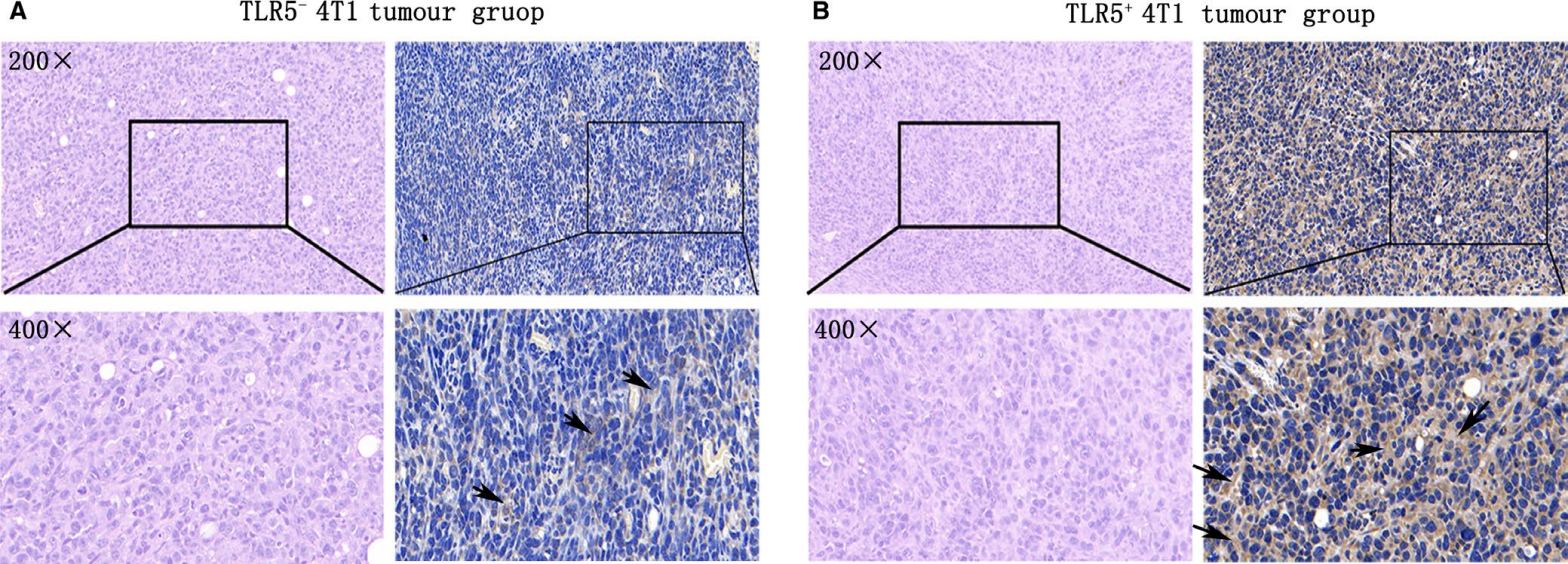
H&E staining and immunohistochemistry staining of ex vivo tumour tissue. TLR5^−^ 4T1 tumours(n = 5), representative H＆E staining microscopy images (left) at 200× and 400× magnification and TLR5 immunohistochemistry staining (right), black arrow refers to the positive area (brown) (A). TLR5^+^ tumours (n = 5), representative H＆E staining microscopy images (left) at 200× and 400× magnification and TLR5 immunohistochemistry staining (right), black arrow refers to the positive area (brown) (B). The data are presented from three independent experiments

For TLR5^−^ and TLR5^+^ 4T1 tumours, representative microscopy images at 200× and 400× magnification from the HE staining (left) and immunohistochemistry staining for TLR5 (right) are shown in Figure 5A and 5. For H＆E staining, they both showed similar tumour tissue and almost no difference was found between them. For immunohistochemistry staining, the positive brown areas were found on the cell membrane and plasma, and the percentage of positive staining of TLR5^+^ is 69.75 ± 5.25%, much higher than that in TLR5^−^ tumours (21.75 ± 3.15%), n = 5, ^***^ *P* < .01.

## 4 DISCUSSION

The promising era of cancer diagnosis is greatly developed through the targeted molecules finding.^8^, ^26^ However, TNBC’s targeted therapy cannot be performed until now since its absence of PR, ER and HER2 expression. So, searching for novel target tightly co‐related with progression of TNBC is of great significance for its diagnosis and therapy.

Recently, it was reported that TLR5 was associated with tumour growth and metastasis,^27^ and TLR5 expression was positively related with allo‐transplant rejection in our previously study.^24^ Considering of TLR5 expressed on NK cells within breast cancer reported by other research^28^ and TLR5 expression is able to restrain tumour growth and metastasis both in vitro and in vivo,^29^ we postulated that TLR5 on TNBC cells may play an important role in TNBC progression, which may be a new target suitable for diagnosis and targeted therapy for TNBC.

Here, firstly we proved that TNBC cell line 4T1 expressed TLR5 both on mRNA and protein level; then, we constructed a TLR5 known down 4T1 cell line (TLR5^−^ 4T1) with lentivirus‐shRNA TLR5 transfection, which is stable technique in down‐regulation a definite gene expression. Our results proved that with this treatment, TLR5 expression was indeed down‐regulated both on mRNA and protein level. Since TNBC has no targeting molecules reported until now, we prepared a radio‐iodine 125 labelled anti‐TLR5 tracer which showed higher labelled rate and high affinity to TLR5 on 4T1 cell in vitro, and relatively stable in serum and NS.

Further, we investigated that whether TLR5 could be used in targeting molecule imaging for TNBC in vivo. ^125^I‐antiTLR5 mAb was injected into 4T1 tumour model mice to evaluate specificity of targeted probe based on TLR5 expression in tumour models, and the results revealed much higher radiotracer retention in 4T1 (TLR5^+^) tumours than in 4T1 (TLR5^−^) tumours at all checking time‐points both ex vivo distribution and in vivo whole‐body phosphor‐autoradiography. In block group with unlabelled anti‐TLR5 antibody pre‐treatment, ^125^I‐antiTLR5 mAb failed to target TLR5 in tumour‐bearing mice, no tumour radio image could be obtained, which revealed the specificity of the ^125^I‐antiTLR5 mAb imaging. ^125^I‐IgG is isotype control to show non‐specific imaging of Fc fragment. ^125^I‐IgG failed to target TLR5 accurately in tumour‐bearing mice, exhibiting non‐specific retention in tumours. Using fluorescent reporter gene markers to detect the growth, migration labelled cells in living organisms is applied in many kinds of diseases in vivo.^30^ Here, we used this technology to monitor the location of 4T1 tumour, and the results suggested that fluorescence imaging of 4T1 tumours in both TLR5 knock‐down and negative control virus transfection was much clearer than no virus transfected 4T1 tumour, and with advantage for clearly tumour edge displaying. And more importantly, we found that the locations of fluorescence were tightly consisted with tracer radioactivity, which further proved that tumour image was TLR5 expression image specificity.

These data suggest that TLR5 should be considered as an in vivo biomarker of TNBC. It could be applied for non‐invasive molecular imaging to monitor tumour development and metastasis, and evaluate therapy response, even as a target for therapy. Low uptake of tracer in main organs such as the heart and high uptake in tumours apparently increased image contrast. These tumour cell‐specific and favourable non‐target clearance features of ^125^I‐antiTLR5 mAb make it a promising radiotracer for TNBC imaging in vivo.

Targeted radioimmunoimaging with monoclonal antibody has been considered a successful imaging for many kinds of cancer.^10^ Radiolabelled ^125^I‐antiTLR5 mAb may provide a method for visualizing TLR5 expression in vivo. Our data suggested that ^125^I‐antiTLR5 mAb could be used in these TNBC tumour models, which is more clinically relevant.

However, there are some limitations for this study: The Molecular 125 weight of monoclonal antibodies is large and it metabolizes slowly in the human body. So it isn't well suitable for clinical application. To improve clinical application, the optimized choice is to reduce extended circulation through selecting antibody fragment or adapter or other small size molecules specifically binding to TLR5, and also we need further study for TLR5 targeting probe with different radioisotope labelled suitable for clinical application, such as iodine 131 or positron nuclide F‐18. Another question is the molecular mechanism underlying regulation of TLR5 in different histological subtype of breast cancer. So, further in vitro and in vivo investigations about TLR5 in breast cancer are required.

In conclusion, TLR5 expression on TNBC cell line 4T1 stable was knocked down successfully. ^125^I‐antiTLR5 mAb was successfully prepared; in vitro and in vivo experiments results revealed that TLR5 expressed on the surface of 4T1 cells was a specific imaging target of ^125^I‐antiTLR5 mAb; TLR5 is a new reporter for triple‐negative breast cancer non‐invasive imaging. Our results supply a new strategy for TLR5‐positive tumour monitoring.

## CONFLICT OF INTEREST

The authors confirm that there are no conflicts of interest.

## AUTHOR CONTRIBUTIONS

Guihua Hou designed research; Dai Shi conducted research; Dai Shi analysed date; Guihua Hou, Dai Shi, Weiwei Liu and Shanshan Zhao wrote the paper; and all members had primary responsibility for final content. All the authors read and approved the final manuscript.

## Data Availability

The data that support the findings of this study are openly available in [repository name eg ‘figshare’] at http://doi.org/[doi], reference number [reference number].
